# Acesso a medicamentos para o tratamento de hipertensão arterial sistêmica e diabetes mellitus tipo 2 na população brasileira: dados da *Pesquisa Nacional de Saúde* de 2019

**DOI:** 10.1590/0102-311XPT241022

**Published:** 2024-09-16

**Authors:** Adriana Amorim de Farias Leal, Maria Helena Rodrigues Galvão, Ângelo Giuseppe Roncalli

**Affiliations:** 1 Universidade Federal do Rio Grande do Norte, Natal, Brasil.; 2 Centro Universitário Unifacisa, Campina Grande, Brasil.

**Keywords:** Doença Crônica, Hipertensão, Diabetes Mellitus, Acessibilidade aos Serviços de Saúde, Acesso a Medicamentos Essenciais, Chronic Disease, Hypertension, Diabetes Mellitus, Health Services Accessibility, Access to Essential Medicines, Enfermedad Crónica, Hipertensión, Diabetes Mellitus, Accesibilidad a los Servicios de Salud, Acceso a los Medicamentos Esenciales

## Abstract

Este estudo objetivou mensurar o acesso aos medicamentos para o tratamento da hipertensão arterial sistêmica e diabetes mellitus tipo 2 no Brasil segundo a via de obtenção, bem como analisar os fatores associados a esse acesso, de acordo com os dados da *Pesquisa Nacional de Saúde* (PNS) de 2019. Foram analisados dados socioeconômicos e relacionados ao uso de medicamentos de pessoas de 15 anos ou mais, em relação ao acesso via Programa Farmácia Popular do Brasil (PFPB) e via serviço público. A maior parte dos brasileiros que participaram da PNS referiu fazer uso do medicamento para controle da hipertensão, nos últimos 15 dias (91,5%), assim como a maior parte referiu fazer uso de medicamento oral para diabetes (95,2%) e/ou uso da insulina (70%).Os medicamentos orais para hipertensão arterial sistêmica e diabetes mellitus tipo 2 foram obtidos majoritariamente via PFPB, sendo respectivamente (45,2% e 53,6%), e os fatores que mais influenciaram negativamente esse acesso foram maior faixa etária, menor renda, menor escolaridade, não ter plano de saúde e referir uma autoavaliação de saúde muito ruim. O acesso à insulina, por sua vez, se deu com maior frequência via serviço público de saúde (69,7%), e os fatores que mais influenciaram negativamente esse acesso foram raça preta/parda, menor renda, não ter plano de saúde e referir uma autoavaliação de saúde muito ruim. De forma geral, foi evidenciada a importância do PFPB como política de ampliação de acesso a medicamentos essenciais no Brasil, considerando a gratuidade dos anti-hipertensivos e antidiabéticos.

## Abstract (en)

This study aimed to measure access to medicines for the treatment of systemic arterial hypertension and type 2 diabetes mellitus in Brazil according to the mode of acquisition, as well as to analyze the factors associated with this access, based on data from the 2019 *Brazilian National Survey of Health* (PNS, acronym in Portuguese). Socioeconomic data and data related to the use of medicines by people aged 15 and over were analyzed in relation to access via the Brazilian Popular Pharmacy Program (PFPB, acronym in Portuguese) and via public services. The majority of Brazilians who took part in the PNS reported using medication to control hypertension in the previous 15 days (91.5%) and using oral medication for diabetes (95.2%) and/or insulin (70%). Most participants obtained oral medication for hypertension and type 2 diabetes mellitus via PFPB (45.2% and 53.6%, respectively), and the factors that most negatively influenced this access were older age, lower income, lower schooling, very poor self-rated health and not having private health insurance. Access to insulin, on the other hand, was most often via the public health service (69.7%), and the factors that most negatively influenced this access were black/mixed-race skin color, lower income, very poor self-rated health and not having private health insurance. Generally, the importance of the PFPB as a policy to increase access to essential medicines in Brazil was highlighted, considering the free supply of antihypertensive and antidiabetic drugs.

## Resumen

Este estudio tuvo como objetivo medir el acceso a los medicamentos para el tratamiento de la hipertensión arterial sistémica y de la diabetes mellitus tipo 2 en Brasil según la vía de obtención, además de analizar los factores asociados a este acceso, según datos de la *Encuesta Nacional de Salud* (PNS) de 2019. Se analizaron datos socioeconómicos y relacionados con el uso de medicamentos de personas de 15 años o más, con relación al acceso por medio del Programa Farmacia Popular de Brasil (PFPB) y por medio del servicio público. La mayor parte de los brasileños que participaron en la PNS refirió utilizar medicamentos para controlar la hipertensión, en los últimos 15 días (91,5%), así como la mayoría refirió el uso de medicamentos orales para la diabetes (95,2%) o uso de insulina (70%). Los medicamentos orales para hipertensión arterial sistémica y diabetes mellitus tipo 2 se obtuvieron en su mayoría por medio del PFPB, respectivamente (45,2% y 53,6%), y los factores que influyeron de forma más negativa en este acceso fueron mayor rango de edad, menores ingresos, menor escolaridad, no tener seguro de salud y reportar una autoevaluación de salud muy mala. El acceso a la insulina, a su vez, se produjo con mayor frecuencia por medio del servicio público de salud (69,7%), y los factores que influyeron de forma más negativa en este acceso fueron la raza negra/morena, menores ingresos, no tener plan de salud y reportar una autoevaluación de salud muy mala. En general, se destacó la importancia de la PFPB como política de ampliación del acceso a medicamentos esenciales en Brasil, considerando la gratuidad de los antihipertensivos y antidiabéticos.

## Introdução

No mundo, as doenças crônicas não transmissíveis (DCNT) são responsáveis pela maior carga de morbimortalidade, acarretando perda de qualidade de vida, limitações, incapacidades, além de alta taxa de mortalidade prematura (entre 30 e 69 anos) ^1^ ^,^ ^2^. No Brasil, de acordo com o Plano de Ações Estratégicas para o Enfrentamento das DCNT de 2011 a 2022, a hipertensão arterial sistêmica e o diabetes mellitus tipo 2 constituíram as causas centrais de morbimortalidade no país, com elevadas repercussões sociais e econômicas ^3^. Em 2019, as DCNT foram responsáveis por 73% dos óbitos ^3^ ^,^ ^4^.

Diante disso, as DCNT, ao lado das doenças infectocontagiosas e das causas externas, contribuem para o cenário da tripla carga de doenças no Brasil e, por isso, importantes políticas públicas para sua prevenção e controle têm sido implementadas, entre elas a ampliação das políticas de acesso a medicamentos ^4^ ^,^ ^5^.

O Sistema Único de Saúde (SUS) atualmente assegura o acesso a medicamentos para o tratamento de DCNT na atenção primária à saúde (APS), nas farmácias básicas da Estratégia Saúde da Família (ESF), com base no elenco de medicamentos atualizado a cada dois anos pela Relação Nacional de Medicamentos Essenciais (Rename) ^6^ ^,^ ^7^. Dessa forma, pode-se estabelecer que o acesso a medicamentos no Brasil se dá por três vias: de forma equitativa e gratuita por meio do SUS; pagamento direto do próprio cidadão nas farmácias privadas; e na forma de copagamento pelo Programa Farmácia Popular do Brasil (PFPB) ^8^ ^,^ ^9^.

Paralelamente, o PFPB, desde 2004, visa ofertar uma assistência farmacêutica integral, não necessariamente de forma gratuita, ao ampliar o acesso a medicamentos de uma parcela da população que pode arcar com o copagamento. Embora desde 2017 não conte mais com sua rede própria de farmácias, o programa continua por meio da iniciativa Aqui Tem Farmácia Popular, em que as farmácias privadas aderem ao programa. A partir de 2011, tornou-se gratuito o acesso para os anti-hipertensivos e antidiabéticos, além dos medicamentos para o tratamento da asma ^8^ ^,^ ^9^.

Diante desse cenário, os inquéritos populacionais auxiliam na compreensão do panorama de acesso a medicamentos. No Brasil, os dados mais recentes sobre acesso a medicamentos são provenientes da *Pesquisa Nacional de Saúde* (PNS), em sua segunda edição feita em 2019, cujo principal objetivo é fornecer informações sobre os determinantes, condicionantes e necessidades de saúde da população brasileira, permitindo estabelecer medidas consistentes, capazes de auxiliar na elaboração de políticas públicas e alcançar maior efetividade nas intervenções em saúde ^10^.

Dessa forma, o objetivo deste artigo foi mensurar o acesso aos medicamentos para o tratamento da hipertensão arterial sistêmica e da diabetes mellitus tipo 2 (oral e/ou insulina) no Brasil segundo a via de obtenção, bem como analisar os fatores associados a esse acesso, conforme os dados da PNS 2019.

## Material e métodos

### Desenho do estudo

Trata-se de estudo transversal desenvolvido com dados da PNS 2019, conduzida pelo Instituto Brasileiro de Geografia e Estatística (IBGE) em parceria com o Ministério da Saúde. A PNS, que teve sua primeira edição em 2013, é uma pesquisa domiciliar de abrangência nacional, representativa de residentes em domicílios particulares permanentes, pertencentes às áreas tanto urbanas quanto rurais, tendo como principal objetivo avaliar, com uma periodicidade quinquenal, o desempenho do sistema nacional de saúde e das condições de vida e saúde da população brasileira, com a perspectiva de subsidiar o planejamento e a avaliação de políticas públicas de saúde para o SUS ^10^.

**Figura 1:**
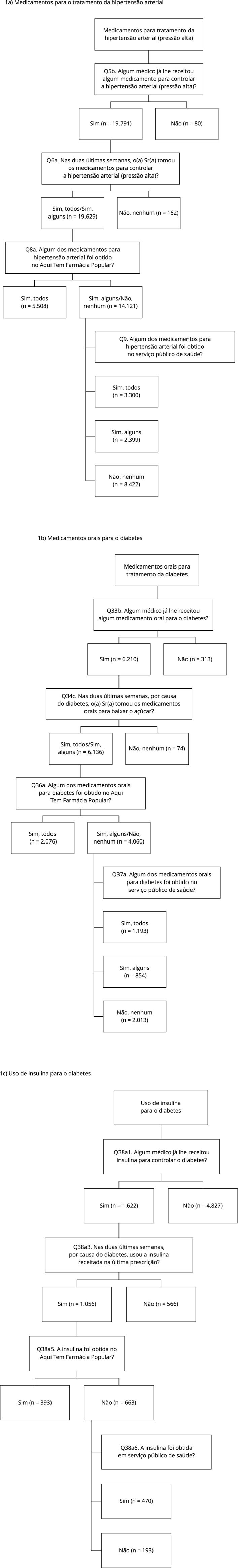
Fluxograma de obtenção da amostra, considerando as respostas da *Pesquisa Nacional de Saúde* (PNS) de 2019 sobre medicamentos para o tratamento da hipertensão arterial, medicamentos orais para o diabetes e uso de insulina para o diabetes. Brasil, 2019.

O módulo Q da PNS 2019, referente às DCNT, foi respondido de forma individual pelo morador sorteado no terceiro estágio amostral da PNS 2019. Sendo assim, as análises deste artigo foram feitas com a amostra de indivíduos de 15 anos ou mais, considerando a seguinte sequência de respostas sobre medicamentos para o tratamento da hipertensão arterial, medicamentos orais para o diabetes e insulina para o diabetes: se teve prescrição do medicamento; se usou o medicamento prescritos; se obteve o medicamento via PFPB; e se obteve o medicamento via serviço público de saúde. A Figura 1 apresenta os fluxogramas de obtenção da amostra.

Fonte: PNS 2019 ^10^. Nota: o motivo de não uso dos medicamentos foi investigado e suas categorias constam na seção *Materiais e Métodos*.

### Variáveis

Foram consideradas variáveis dependentes do acesso a medicamentos prescritos para o tratamento de hipertensão arterial sistêmica e/ou diabetes mellitus tipo 2 (oral e/ou insulina) de acordo com as duas principais fontes de acesso pesquisadas pelo PNS (questão J33a. Algum dos medicamentos foi obtido no programa Aqui Tem Farmácia Popular?) e pelo serviço público (questão J34. Algum dos medicamentos foi obtido em serviço público de saúde?).

De forma independente, foram avaliadas variáveis sociodemográficas e de condições de saúde, incluindo faixa etária em anos (15-29; 30-59; 60-79; 80 ou mais), sexo (feminino; masculino), raça/cor da pele (branca; preta; amarela; parda; indígena), renda familiar *per capita* (em Reais) por quintis (5º quintil - ≥ 2.097,00); 4º quintil - 1.249,00-2.096,00; 3º quintil - 1.000,00-1.248,00); 2º quintil - 547,00-999,00); 1º quintil - ≤ 546,00), escolaridade (Superior completo; Superior incompleto; Médio completo; Médio incompleto; Fundamental completo; Fundamental incompleto; sem instrução), ocupação (pessoas ocupadas; pessoas desocupadas; pessoas fora da força de trabalho), autoavaliação de saúde (muito bom; bom; regular; ruim; muito ruim), possui plano de saúde (sim; não), domicílio cadastrado na ESF (sim; não sabe; não), área geográfica (urbana; rural), região de residência (Sudeste; Norte; Nordeste; Sul; Centro-oeste).

Em relação às variáveis relacionadas ao uso de medicamentos prescritos para o tratamento de hipertensão arterial sistêmica e/ou diabetes mellitus tipo 2 (oral e/ou insulina) em questão, foram incluídas: uso do medicamento e/ou insulina nos últimos 15 dias (sim, todos; sim, alguns; não, nenhum); principal motivo para ter tomado os medicamentos receitados (não conseguiu obter no serviço público de saúde; não conseguiu o(s) medicamento (s) no Aqui Tem Farmácia Popular; a farmácia era distante ou teve dificuldade de transporte; não conseguiu encontrar todos os medicamentos para comprar na farmácia; não tinha dinheiro para comprar; não achou necessário; não precisa mais tomar medicamentos porque a doença está controlada; outro), obteve algum dos medicamentos no PFPB (sim; não), e obteve algum dos medicamentos no serviço público (sim; não).

### Métodos estatísticos

Foi feita uma análise descritiva, caracterizando os participantes quanto a variáveis sociodemográficas e relacionadas às condições de saúde, com as estimativas para a população brasileira, bem como foram analisadas as estimativas do uso de medicamentos prescritos para o tratamento da hipertensão arterial sistêmica e/ou diabetes mellitus tipo 2 (oral e/ou insulina) e o acesso a esses medicamentos por meio políticas públicas (PFPB e serviço público).

Foram estimadas as razões de prevalência (RP) sobre o acesso a esses medicamentos via PFPB e via serviço público de saúde, segundo as variáveis independentes detalhadas anteriormente. As análises foram feitas usando o software SPSS, versão 20.0 (https://www.ibm.com/).

O modelo de regressão de Poisson foi usado para estimar as RP brutas e ajustadas e intervalos de 95% de confiança (IC95%); na avaliação da significância estatística das diferenças entre os grupos, foi considerado o nível de significância de 5%. Para definir o modelo final, foi desenvolvida uma estratégia *backward*, em que todas as variáveis de interesse foram testadas individualmente e introduzidas no modelo, sendo retiradas uma a uma até que apenas variáveis com significância maior mais de 95% permanecessem.

### Considerações éticas

Os dados da PNS são públicos e estão disponíveis no *site* do IBGE (http://www.ibge.gov.br). A PNS 2019 atendeu a todos os requisitos em pesquisas que envolvem seres humanos e foi aprovada pelo Comitê Nacional de Ética em Pesquisa (protocolo nº 3.529.376).

## Resultados

As caracterizações dos participantes com estimativas para a população brasileira estão contidas na Tabela 1. A maior parte da população com prescrição medicamentosa para o tratamento da hipertensão arterial sistêmica e/ou diabetes mellitus tipo 2 (oral e/ou insulina) são pessoas idosas com idade de 60 a 79 anos (46,8%), mulheres (58,3%), pessoas que referiram raça/cor da pele branca (45,8%), residentes na Região Sudeste do Brasil (47,8%) e que residiam em domicílios cadastrados na ESF (63,4%).

**Tabela 1:** Características sociodemográficas dos adultos brasileiros com prescrição de medicamento para tratamento da hipertensão arterial sistêmica e/ou diabetes mellitus tipo 2 (oral e/ou insulina), segundo a *Pesquisa Nacional de Saúde*. Brasil, 2019.

Características	n	% (IC95%)
Faixa etária (anos)		
15-29	275.221	1,4 (1,2-1,6)
30-59	8.552.135	43,3 (41,9-44,9)
60-79	9.251.935	46,8 (45,6-48,0)
80 ou mais	1.690.151	8,5 (8,0-9,2)
Sexo		
Feminino	11.523.168	58,3 (57,3-59,3)
Masculino	8.246.275	41,7 (40,7-42,7)
Raça/Cor da pele		
Branca	9.049.838	45,8 (44,7-46,8)
Preta	2.409.748	12,2 (11,5-12,9)
Amarela	229.256	1,2 (0,9-1,4)
Parda	7.959.450	40,3 (39,3-41,2)
Indígena	121.150	0,6 (0,5-0,8)
Renda familiar *per capita* em Reais (quintis)		
5º (≥ 2.097,00)	4.284.959	21,7 (20,6-22,8)
4º (1.249,00-2.096,00)	4.398.560	22,3 (21,4-23,1)
3º (1.000,00-1.248,00)	2.139.162	10,8 (10,2-11,5)
2º (547,00-999,00)	5.648.182	28,6 (27,6-29,6)
1º (≤ 546,00)	3.297.421	16,7 (16,0-17,4)
Escolaridade		
Superior completo	2.432.301	12,3 (11,5-13,1)
Superior incompleto	426.745	2,2 (1,9-2,5)
Médio completo	3.747.722	19,0 (18,1-19,8)
Médio incompleto	657.161	3,3 (3,0-3,7)
Fundamental completo	1.652.499	8,4 (7,8-9,0)
Fundamental incompleto	8.504.160	43,0 (41,9-44,1)
Sem instrução	2.348.853	11,9 (11,3-12,5)
Ocupação		
Pessoas ocupadas	8.031.494	40,6 (39,6-41,6)
Pessoas desocupadas	424.213	2,1 (1,8-2,5)
Pessoas fora da força de trabalho	11.313.736	57,2 (56,2-58,3)
Autoavaliação de saúde		
Muito bom	2.038.477	10,3 (9,7-11,0)
Bom	9.995.661	50,6 (49,5-51,6)
Regular	6.179.051	31,3 (30,2-32,3)
Ruim	1.233.641	6,2 (5,8-6,7)
Muito ruim	322.610	1,6 (1,4-1,9)
Possui plano de saúde		
Sim	5.609.775	28,4 (27,3-29,5)
Não	14.159.668	71,6 (70,5-72,7)
Domicílio cadastrado na ESF		
Sim	12.533.161	63,4 (62,0-64,8)
Não sabe	5.349.989	27,1 (25,8-28,4)
Não	19.769.444	9,5 (8,9-10,2)
Área geográfica		
Urbana	17.169.747	86,8 (86,3-87,4)
Rural	2.599.696	13,2 (12,6-13,7)
Região de residência		
Sudeste	9.453.247	47,8 (46,7-49,0)
Norte	997.396	5,0 (4,8-5,6)
Nordeste	4.901.446	24,8 (24,0-25,6)
Sul	3.065.173	15,5 (14,9-16,2)
Centro-oeste	1.352.209	6,8 (6,5-7,2)

ESF: Estratégia Saúde Família; IC95%: intervalo de 95% de confiança.

A maior parte dos brasileiros fez uso de medicamento prescrito para controle da hipertensão nos últimos 15 dias (91,5%). Dentre as pessoas que afirmaram não tomar o medicamento, a maior parte não tomou devido à pressão arterial estar controlada no período (45,8%) ou por não acharem necessário (38,3%). Grande parte dos entrevistados referiu ter acesso aos medicamentos para tratamento da hipertensão a partir do Aqui Tem Farmácia Popular (45,2%), com exceção dos que tiveram acesso exclusivamente pelo programa, 41% tiveram acesso por meio do serviço público. A frequência de uso de medicamento oral para diabetes nos últimos 15 dias foi de 95,2%. Os principais motivos para não usar os medicamentos também foram devido a diabetes estar controlada no período (42,5%) e por não achar necessário (36,5%). A maior parte obteve algum dos medicamentos orais para diabetes por meio do Aqui Tem Farmácia Popular (53,6%), enquanto, dentre os que não obtiveram todos os medicamentos pelo programa, 41% tiveram acesso pelo serviço público (Tabela 2).

**Tabela 2:** Estimativas do uso de medicamentos prescritos para o tratamento da hipertensão arterial sistêmica e/ou diabetes mellitus tipo 2 (oral e/ou insulina), em relação ao acesso a esses medicamentos por políticas públicas, segundo a *Pesquisa Nacional de Saúde*. Brasil, 2019.

Variáveis	Uso dos medicamentos prescritos para tratamento da hipertensão arterial sistêmica		Uso dos medicamentos orais para tratamento do diabetes mellitus tipo 2		Uso da insulina para tratamento do diabetes mellitus tipo 2	
	n	% (IC95%)	n	% (IC95%)	n	% (IC95%)
Tomou o medicamento prescrito nos últimos 15 dias						
Sim, todos	16.315.896	91,5 (90,9-92,2)	5.178.853	95,2 (94,2-96,0)	1.020.949	70,0 (66,5-73,2)
Sim, alguns	188.685	1,1 (0,9-1,3)	89.871	1,7 (1,2-2,2)	*	*
Não, nenhum	1.319.625	7,4 (6,8-8,1)	173.744	3,2 (2,58-4,0)	438.112	30,0 (26,8-33,5)
Principal motivo de não ter tomado os medicamentos prescritos						
Não conseguiu obter no serviço público de saúde	56.196	3,7 (2,8-4,9)	22.864	8,7 (5,0-14,6)	6.771	1,5 (0,8-3,1)
Não conseguiu o(s) medicamento(s) no Aqui Tem Farmácia Popular	10.813	0,7 (0,3-2,0)	7.662	2,6 (0,8-10,3)	*	*
A farmácia era distante ou teve dificuldade de transporte	7.359	0,5 (0,2-1,0)	1.193	0,5 (0,1-1,5)	1.593	0,4 (0,1-1,1)
Não conseguiu encontrar todos os medicamentos para comprar na farmácia	7.068	0,5 (0,2-1,0)	686	0,3 (0,1-1,3)	695	0,2 (0,0-0,7)
Não tinha dinheiro para comprar	44.644	3,0 (2,0-4,5)	4.344	1,6 (0,7-4,0)	4.098	0,9 (0,4-2,3)
Não achou necessário	578.379	38,3 (34,0-42,8)	96.261	36,5 (28,2-45,7)	84.102	19,2 (14,4-25,2)
Não precisa mais tomar medicamentos porque a doença está controlada	690.294	45,8 (41,6-50,0)	112.121	42,5 (33,2-52,4)	306.151	69,9 (63,5-75,6)
Outro	113.554	7,5 (5,6-10,1)	18.481	7,0 (3,8-12,7)	34.701	7,9 (4,9-12,6)
Obteve algum dos medicamentos no PFPB						
Sim	7.467.106	45,2 (43,8-46,7)	2.826.180	53,6 (51,6-55,6)	435.680	42,7 (38,3-47,2)
Não	9.037.475	54,8 (53,3-56,2)	2.442.544	46,4 (44,4-48,4)	585.268	57,3 (52,8-61,7)
Obteve algum dos medicamentos para hipertensão no serviço público						
Sim	4.621.110	41,0 (39,6-42,3)	4.621.110	41,0 (39,6-42,3)	408.038	69,7 (64,2-74,7)
Não	6.662.637	59,0 (57,7-60,4)	6.662.637	59,0 (57,7-60,4)	177.229	30,3 (25,3-35,8)

IC95%: intervalo de 95% de confiança; PFPB: Programa Farmácia Popular do Brasil.

* Não houve esse questionamento em relação ao uso da insulina.

Com relação ao uso da insulina receitada na última prescrição, 70% referiram ter utilizado. Dentre os que não utilizaram, a maioria não o fez pois a diabetes estava controlada sem o uso da insulina (69,9%) ou não achou necessário (19,2%). Ao considerar o acesso à insulina por políticas públicas, 42,7% referiram acessá-la pelo PFPB. Dentre os que não tiveram acesso exclusivamente por esse programa, 69,7% tiveram acesso à insulina por meio do serviço público (Tabela 2).

Estiveram associadas ao maior acesso aos medicamentos para tratamento da hipertensão arterial sistêmica arterial via PFPB a faixa etária acima de 60 anos, menor renda, menor escolaridade e ausência de plano de saúde privado. Uma maior prevalência de acesso ao medicamento de hipertensão pelo mesmo programa foi associada à ausência de cobertura na APS e a residir em área rural ou nas regiões Norte e Nordeste. Com relação ao acesso ao medicamento para hipertensão pelo serviço público, uma maior prevalência foi observada entre pessoas pretas ou pardas, com menor renda, com pior autoavaliação de saúde, que não têm plano de saúde. Uma menor prevalência de acesso a medicamentos de hipertensão pelo serviço público esteve associada a ser do sexo masculino, pessoas que residem em domicílios não cadastrados na APS e residentes nas regiões Norte, Nordeste e Centro-oeste (Tabela 3).

**Tabela 3:** Fatores associados ao acesso a medicamentos prescritos para tratamento da hipertensão arterial sistêmica, segundo a via de obtenção, a partir do modelo de regressão múltipla de Poisson, segundo a *Pesquisa Nacional de Saúde*. Brasil, 2019.

Características	Obtenção via PFPB		Obtenção via serviço público	
	RP ajustada (IC95%)	Valor de p	RP ajustada (IC95%)	Valor de p
Faixa etária (anos)				
15-29	1,00		-	-
30-59	2,09 (1,45-3,02)	< 0,001	-	-
60-79	2,07 (1,43-2,99)	< 0,001	-	-
80 ou mais	1,70 (1,17-2,47)	0,005	-	-
Sexo				
Feminino	-	-	1,00	-
Masculino	-	-	0,93 (0,88-0,99)	0,018
Raça/Cor da pele				
Branca	-	-	1,00	
Preta	-	-	1,12 (1,03-1,22)	0,010
Amarela	-	-	1,04 (0,75-1,45)	0,814
Parda	-	-	1,10 (1,02-1,17)	0,008
Indígena	-	-	1,09 (0,78-1,52)	0,600
Renda familiar *per capita* em Reais (quintis)				
5º (≥ 2.097,00)	1,00	-	1,00	-
4º (1.249,00-2.096,00)	1,10 (1,01-1,20)	0,037	1,49 (1,30-1,72)	< 0,001
3º (999,00-1.248,00)	1,20 (1,09-1,32)	< 0,001	1,62 (1,39-1,88)	< 0,001
2º (547,00-999,00)	1,21 (1,10-1,31)	< 0,001	1,67 (1,46-1,92)	< 0,001
1º (≤ 546,00)	1,19 (1,08-1,31)	0,001	1,90 (1,65-2,19)	< 0,001
Escolaridade				
Superior completo	1,00	-	-	-
Superior incompleto	1,15 (0,92-1,43)	0,215	1,44 (1,06-1,96)	
Médio completo	1,24 (1,10-1,39)	0,001	1,36 (1,11-1,66)	
Médio incompleto	1,35 (1,17-1,57)	< 0,001	1,37 (1,06-1,77)	
Fundamental completo	1,31 (1,15-1,49)	< 0,001	1,69 (1,37-2,09)	
Fundamental incompleto	1,35 (1,20-1,51)	< 0,001	1,73 (1,42-2,10)	
Sem instrução	1,26 (1,10-1,45)	0,001	1,67 (1,36-2,04)	
Autoavaliação de saúde				
Muito bom	-	-	-	-
Bom	-	-	1,33 (1,15-1,55)	< 0,001
Regular	-	-	1,46 (1,25-1,70)	< 0,001
Ruim	-	-	1,65 (1,39-1,96)	< 0,001
Muito ruim	-	-	1,58 (1,28-1,96)	< 0,001
Possui plano de saúde				
Sim	1,00		1,00	
Não	1,16 (1,08-1,24)	< 0,001	1,77 (1,58-1,98)	< 0,001
Domicílio cadastrado na ESF				
Sim	1,00	-		
Não sabe	0,88 (0,80-0,97)	0,007	0,79 (0,70-0,85)	< 0,001
Não	0,92 (0,86-0,98)	0,008	0,77 (0,70-0,85)	< 0,001
Área geográfica				
Urbana	1,00	-	-	-
Rural	0,83 (0,77-0,90)	< 0,001	-	-
Região de residência				
Sudeste	1,00	-	-	-
Norte	0,75 (0,68-0,83)	< 0,001	0,75 (0,68-0,82)	< 0,001
Nordeste	0,69 (0,63-0,74)	< 0,001	0,76 (0,70-0,82)	< 0,001
Sul	1,12 (1,05-1,20)	0,001	1,23 (1,13-1,34)	< 0,001
Centro-oeste	0,98 (0,89-1,07)	0,614	0,86 (0,78-0,96)	0,005

ESF: Estratégia Saúde da Família; IC95%: intervalo de 95% de confiança; PFPB: Programa Farmácia Popular do Brasil; RP: razão de prevalência.

Ao avaliar os fatores associados ao acesso a medicamentos orais para diabetes por meio do Aqui Tem Farmácia Popular, observou-se uma maior prevalência de acesso entre as pessoas com menor renda e que não contam com plano de saúde. Enquanto pessoas que residiam nas regiões Norte e Nordeste tiveram uma menor prevalência de acesso ao medicamento oral para diabetes por meio do mesmo programa. Com relação ao acesso a medicamentos orais para diabetes a partir do serviço público, uma maior prevalência esteve associada a uma menor renda e uma pior autoavaliação de saúde. Por outro lado, uma menor prevalência de acesso a medicamentos orais para diabetes pelo serviço público esteve associada a uma maior faixa etária, residir em um domicílio não cadastrado na APS e residir nas regiões Norte e Nordeste do país (Tabela 4).

**Tabela 4:** Fatores associados ao acesso de medicamentos orais para tratamento de diabetes mellitus tipo 2, segundo a via de obtenção, a partir de modelos de regressão múltipla de Poisson, segundo a *Pesquisa Nacional de Saúde*. Brasil, 2019.

Características	Obtenção via PFPB		Obtenção via serviço público	
	RP ajustada (IC95%)	Valor de p	RP ajustada (IC95%)	Valor de p
Faixa etária (anos)				
15-29	-	-	1,00	-
30-59	-	-	0,82 (0,61-1,10)	0,188
60-79	-	-	0,84 (0,63-1,13)	0,250
80 ou mais	-	-	0,66 (0,47-0,93)	0,017
Sexo				
Feminino	-	-	1,00	-
Masculino	-	-	0,87 (0,80-0,95)	0,002
Renda familiar *per capita* em Reais (quintis)				
5º (≥ 2.097,00)	1,00	-	1,00	-
4º (1.249,00-2.096,00)	0,97 (0,88-1,07)	0,065	1,58 (1,30-1,93)	< 0,001
3º (999,00-1.248,00)	1,18 (1,03-1,35)	0,021	1,57 (1,26-1,95)	< 0,001
2º (547,00-999,00)	1,18 (1,05-1,32)	0,006	1,72 (1,42-2,32)	< 0,001
1º (≤ 546,00)	1,24 (1,09-1,40)	0,001	1,92 (1,59-2,32)	< 0,001
Autoavaliação de saúde				
Ótimo	-	-	1,00	-
Bom	-	-	1,18 (0,95-1,48)	0,142
Regular	-	-	1,25 (1,00-1,58)	0,053
Ruim	-	-	1,36 (1,05-1,75)	0,020
Péssimo	-	-	1,36 (1,01-1,82)	0,040
Possui plano de saúde				
Sim	1,00		1,00	-
Não	1,18 (1,07-1,29)	0,001	1,90 (1,61-2,24)	< 0,001
Domicílio cadastrado na ESF				
Sim	-	-	1,00	
Não sabe	-	-	0,92 (0,77-1,09)	0,317
Não	-	-	0,86 (0,76-0,97)	0,014
Região de residência				
Sudeste	1,00	-	1,00	-
Norte	0,59 (0,51-0,69)	< 0,001	0,68 (0,58-0,80)	< 0,001
Nordeste	0,65 (0,59-0,72)	< 0,001	0,74 (0,67-0,82)	< 0,001
Sul	1,08 (0,99-1,18)	0,076	1,06 (0,94-1,18)	0,337
Centro-oeste	0,97 (0,88-1,07)	0,509	0,87 (0,76-1,01)	0,063

ESF: Estratégia Saúde da Família; IC95%: intervalo de 95% de confiança; PFPB: Programa Farmácia Popular do Brasil; RP: razão de prevalência.

Ao avaliar o acesso à insulina por políticas públicas no Brasil, esteve associado a uma maior prevalência de acesso à insulina por meio do Aqui Tem Farmácia Popular, indígenas e pessoas de cor da pele preta e amarela e pessoas desocupadas ou fora da força de trabalho. Uma menor prevalência de acesso à insulina por esse programa foi observada entre residentes da Região Nordeste. Com relação ao acesso à insulina por meio do serviço público, foi observada uma maior prevalência entre as pessoas de menor renda, cor da pele amarela e que não têm plano de saúde. Uma menor prevalência de acesso à insulina pelo serviço público foi observada em pessoas do sexo masculino e que residiam em domicílios não cadastrados na APS (Tabela 5).

**Tabela 5:** Fatores associados ao acesso à insulina para tratamento de diabetes mellitus tipo 2, segundo a via de obtenção, a partir de modelos de regressão múltipla de Poisson, segundo a *Pesquisa Nacional de Saúde*. Brasil, 2019.

Características	Obtenção via PFPB		Obtenção via serviço público	
	RP ajustada (IC95%)	Valor de p	RP ajustada (IC95%)	Valor de p
Sexo				
Feminino	1,00	-	1,00	-
Masculino	-	-	0,82 (0,71-0,95)	0,008
Ração/Cor da pele				
Branca	1,00	-	1,00	-
Preta	1,52 (1,17-1,97)	0,002	1,00 (0,82-1,22)	0,998
Amarela	1,86 (1,06-3,26)	0,030	2,26 (1,16-1,42)	0,017
Parda	1,06 (0,83-1,35)	0,646	1,01 (0,90-1,14)	0,839
Indígena	3,08 (2,13-4,44)	< 0,001	0,99 (0,80-1,23)	0,929
Ocupação				
Pessoas ocupadas	1,00	-	-	-
Pessoas desocupadas	1,85 (1,28-2,65)	0,001	-	-
Pessoas fora da força de trabalho	1,12 (0,87-1,43)	0,386		
Renda familiar *per capita* em Reais (quintis)				
5º (≥ 2.097,00)	-	-	1,00	-
4º (1.249,00-2.096,00)	-		1,80 (1,18-2,73)	0,006
3º (999,00-1.248,00)	1,91 (1,26-2,89)	0,002		
2º (547,00-999,00)	1,72 (1,15-2,58)	0,008		
1º (≤ 546,00)	-		2,02 (1,36-2,99)	0,001
Possui plano de saúde				
Sim	-		1,00	-
Não	-		1,58 (1,26-1,99)	< 0,001
Domicílio cadastrado na ESF				
Sim	-		1,00	
Não sabe	-		0,79 (0,63-0,99)	0,047
Não	-		0,76 (0,63-0,91)	0,003
Região de residência				
Sudeste	1,00	-	-	-
Norte	0,70 (0,47-1,02)	0,064	-	-
Nordeste	0,65 (0,49-0,86)	0,003	-	-
Sul	1,16 (0,89-1,52)	0,264	-	-
Centro-oeste	0,86 (0,62-1,19)	0,370		

ESF: Estratégia Saúde da Família; IC95%: intervalo de 95% de confiança; PFPB: Programa Farmácia Popular do Brasil; RP: razão de prevalência.

## Discussão

Este artigo mensurou o acesso a medicamentos prescritos para tratamento da hipertensão arterial sistêmica e/ou diabetes mellitus tipo 2 (oral e/ou insulina) de acordo com a fonte pública de obtenção (PFPB ou serviço público de saúde), bem como verificou a associação entre fatores individuais relacionados ao acesso a esses medicamentos, a partir dos resultados da PNS 2019. Com base nesses dados, a maior parte dos brasileiros que participaram da PNS 2019 referiu fazer uso do medicamento para controle da hipertensão arterial sistêmica, nos últimos 15 dias, assim como a maior parte referiu fazer uso de medicamento oral para diabetes mellitus tipo 2 e/ou uso da insulina. De forma geral, a maioria da população brasileira que faz uso de medicamentos prescritos para o tratamento da hipertensão arterial sistêmica e/ou diabetes mellitus tipo 2 (oral e/ou insulina) são mulheres, pessoas idosas com idade de 60 a 79 anos, residentes na Região Sudeste e que residiam em domicílios cadastrados na ESF.

A maior parte dos brasileiros fez uso de medicamentos prescritos para o tratamento de hipertensão arterial sistêmica e/ou diabetes mellitus tipo 2 (oral e/ou insulina), assim como a maioria teve acesso a esses medicamentos via PFPB. Dentre os motivos de não uso desses medicamentos, a maior parte referiu não achar mais necessário ou estar com a doença controlada. Os medicamentos orais prescritos para o tratamento da hipertensão arterial sistêmica e diabetes mellitus tipo 2 foram obtidos majoritariamente via PFPB, e os fatores que mais influenciaram negativamente esse acesso foram: maior faixa etária, menor renda, menor escolaridade, não ter plano de saúde e referir uma autoavaliação de saúde muito ruim. O acesso à insulina prescrita para o tratamento de diabetes mellitus tipo 2, por sua vez, se deu com maior frequência via serviço público de saúde, e os fatores que mais influenciaram negativamente esse acesso foram: raça preta/parda, menor renda, não ter plano de saúde e referir uma autoavaliação de saúde muito ruim.

O perfil de uso de medicamentos para o tratamento de DCNT especialmente em relação à hipertensão arterial sistêmica e ao diabetes mellitus tipo 2, é equivalente ao perfil epidemiológico de países em desenvolvimento como o Brasil, onde há um aumento consubstancial da prevalência e incidência de DCNT e de seus fatores de risco. Além disso, a prevalência de uso em grupos específicos corrobora com os dados sobre acesso a medicamentos de inquéritos populacionais anteriores, e endossa a pluralidade regional brasileira ^5^ ^,^ ^11^ ^,^ ^12^.

O fato da maioria dos residentes na Região Sudeste e em domicílios cadastrados em áreas de abrangência da ESF ter referido o acesso desses medicamentos pelas vias de obtenção em estudo, implica o maior acesso a serviços de saúde e ao diagnóstico médico devido a alocação de recursos e gestão dos serviços de saúde de forma mais eficiente ^13^. Ainda, o tratamento farmacológico, integrado às medidas não farmacológicas, e ofertado sobretudo no nível primário de atenção à saúde figura como uma das formas com melhor relação custo-efetividade para enfrentar as DCNT, principalmente a hipertensão e a diabetes ^14^ ^,^ ^15^.

Da mesma forma, mulheres e idosos buscam com mais frequência os serviços de saúde, implicando em um diagnóstico mais efetivo e em uma maior chance de continuidade da farmacoterapia e, consequentemente, com maiores cuidados à saúde ^16^.

A maior prevalência de acesso a medicamentos orais para o tratamento da diabetes mellitus tipo 2 foi via PFPB. A estratégia de ampliação do acesso, de forma gratuita, a antidiabéticos pelo PFPB garante o cumprimento dos princípios doutrinários do SUS - universalidade, integralidade e equidade, e a assistência terapêutica integral, incluindo a assistência farmacêutica, conforme previstos na *Lei nº 8.080/1990* ^17^. Nesse contexto, os dados da PNS 2013 demonstram que mais da metade (57,4%) dos diabéticos obtiveram pelo menos um medicamento no PFPB, com destaque para a participação dos segmentos socioeconomicamente menos favorecidos ^18^.

O acesso sem desembolso a medicamentos para hipertensão e diabetes a partir do Aqui Tem Farmácia Popular evitou a utilização de serviços de alta complexidade relacionados a essas doenças nos municípios em que o programa foi implementado ^6^. Ainda mais quando se observa o período a partir do qual esses medicamentos passaram a ter distribuição gratuita, sem copagamento, o que aumentou seu uso, indicando que, para muitas pessoas, o preço é uma barreira ao acesso, ainda que esses produtos sejam dispensados em farmácias das unidades de saúde do SUS ^6^.

Assim, os resultados deste estudo sugerem que a melhoria do acesso aos medicamentos pelo referido programa implicou diretamente em um melhor perfil de utilização da farmacoterapia para as DCNT em questão, principalmente para os grupos sociais mais vulneráveis - maior faixa etária, menor renda, menor escolaridade e os que não pagam por plano de saúde, além dos que referiram uma pior situação de saúde, no tocante à sua autopercepção. Isso confirma a importância do acesso aos medicamentos para a obtenção de resultados em saúde melhores, possibilitando maior sobrevida à população e menores custos para o sistema de saúde de forma geral ^19^.

Por outro lado, é importante refletir sobre os motivos de não uso desses medicamentos, quando os usuários referem não achar necessário ou julgarem que a doença está controlada. Tal fato é evidenciado quando se trata de usuários com o diagnóstico de alguma DCNT e pressupõe um trabalho interprofissional de prevenção secundária, pois uma vez com o diagnóstico da doença crônica, a longitudinalidade do cuidado, a adesão à farmacoterapia e a periodicidade nos serviços de saúde, especialmente os da APS, são essenciais para o manejo adequado dessas doenças ^16^ ^,^ ^20^ ^,^ ^21^.

Este estudo mostrou maior prevalência de acesso a pelo menos um dos medicamentos prescritos para tratamento de hipertensão arterial sistêmica e/ou diabetes mellitus tipo 2 por meio do serviço público entre os segmentos da população socialmente mais vulneráveis (pretos ou pardos, menor renda, os que referiram pior autoavaliação de saúde e os sem plano de saúde). O acesso universal a medicamentos pelo setor público permanece um grande desafio, uma vez que grande parcela da população ainda precisa recorrer ao desembolso direto para obter os medicamentos necessários ao seu tratamento, o que compromete significativamente a renda familiar com gastos em saúde e, consequentemente, caracteriza uma penalização à população de menor poder aquisitivo e que necessita do uso contínuo do medicamento ^22^ ^,^ ^23^.

Dados da *Pesquisa Nacional de Acesso e Utilização de Medicamentos* (PNAUM) mostraram que entre os que tiveram acesso total ao tratamento para DCNT, cerca de metade obtiveram todos os seus medicamentos de forma gratuita, uma pequena parte conseguiu algum medicamento gratuito e um terço pagou por todos os medicamentos para tratamento das DCNT ^6^.

Em contrapartida, observou-se que a menor prevalência de acesso a medicamentos prescritos para tratamento da hipertensão arterial sistêmica e/ou diabetes mellitus tipo 2 (orais e/ou insulina) se deu nas regiões mais pobres do país - Norte e Nordeste. Um estudo de Silva et al. ^24^ fez uma análise da equidade da distribuição de recursos do PFPB, e verificou que os estados localizados nas regiões mais ricas são mais assistidos pelo programa do que os estados das regiões mais pobres, Norte e Nordeste. Esse fato pode ser devido a uma questão comercial, uma vez que a expansão e distribuição dos medicamentos ocorre em função da decisão voluntária dos estabelecimentos privados de participar do programa ^24^ ^,^ ^25^.

Ainda que o acesso a medicamentos por meio de políticas públicas seja universal, igualitário e gratuito a todo cidadão brasileiro, e fruto de uma conquista histórica, ainda há dificuldades para tal, pois são visíveis as desigualdades de acesso a medicamentos entre as regiões do Brasil, em especial nas regiões desfavorecidas socioeconomicamente e que, de certa forma, reproduzem a desigualdade socioeconômica brasileira ^23^.

Este estudo utilizou uma amostra representativa da população brasileira, destacando os principais fatores envolvidos com o acesso a medicamentos para o tratamento da hipertensão arterial sistêmica e do diabetes mellitus tipo 2. É importante ressaltar que na versão de 2013 não havia essa questão sobre a prescrição de um medicamento para o tratamento das referidas doenças, e agora, na versão de 2019, foi possível identificar as pessoas que têm o diagnóstico e a prescrição, e que fizeram ou não o uso do medicamento.

Não obstante, é importante indicar como fator limitante os potenciais viés de informação, devido aos dados autorreferidos, o que pode subestimar a prevalência das doenças e viés de memória, principalmente no momento de referir sobre o uso do medicamento e sua fonte de obtenção, além das limitações próprias dos estudos transversais, como o viés de incidência/prevalência.
